# 27-Week avocado-soybean unsaponifiables treatment in a knee osteoarthritis rabbit model: histomorphometric assessment of potential disease-modifying effects

**DOI:** 10.3389/fvets.2026.1821515

**Published:** 2026-05-29

**Authors:** Ana Sabucedo-Suárez, Fernando Muñoz, María Permuy, Silvia Fernández-Martín, Antonio Cantalapiedra-González, Mónica López-Peña

**Affiliations:** 1 Anatomy, Animal Production and Veterinary Clinical Sciences Department, Veterinary Faculty, Universidade de Santiago de Compostela, Lugo, Spain; 2 Laboratory of Biomaterials, Ibonelab S.L., Lugo, Spain

**Keywords:** articular cartilage, avocado-soybean unsaponifiables, histomorphometry, osteoarthritis, rabbit

## Abstract

Osteoarthritis is a chronic degenerative joint disease that affects cartilage and subchondral bone, and there is growing interest in nutraceuticals such as avocado–soybean unsaponifiables as potential disease-modifying agents and possible alternatives to nonsteroidal anti-inflammatory drugs. This study aimed to evaluate the long-term effects of daily oral avocado–soybean unsaponifiables on cartilage and subchondral bone in a surgically induced knee osteoarthritis rabbit model using quantitative histomorphometric, histological and immunohistochemical analyses. Osteoarthritis was induced by anterior cruciate ligament transection plus partial meniscectomy in one knee of adult New Zealand White rabbits, while contralateral knees served as healthy controls and a sham-operated group controlled for surgical manipulation; animals received placebo or avocado–soybean unsaponifiables for 24 weeks before joints were collected for undecalcified and decalcified histology, histomorphometry and collagen type II and type X immunohistochemistry. Osteoarthritic joints showed increased cartilage thickness and surface fibrillation, with a tendency toward subchondral bone thinning beneath affected regions, confirming successful induction of osteoarthritis, whereas trabecular bone parameters and collagen type II expression did not differ significantly between treatment groups, and collagen type X expression increased in osteoarthritic groups compared with sham-operated joints. Long-term avocado–soybean unsaponifiable treatment did not reduce hypertrophic cartilage response, surface fibrillation, trabecular bone loss or collagen remodeling markers, suggesting limited structural efficacy in this model under the evaluated conditions.

## 1 Introduction

Osteoarthritis is a chronic degenerative articular pathology characterized by cellular stress and extracellular matrix (ECM) degradation. Affecting mobile joints and representing one of the most common disability causes, OA causes a significant socioeconomical and life-quality impact of a half of the population over 60 years of age, and beyond its human prevalence, OA has become one of the most frequently diagnosed musculoskeletal conditions in companion animals as well (1).

The pathophysiology of OA involves a complex interplay of mechanisms, including fibrosis of periarticular tissues (ligaments, tendons, menisci, joint capsules), progressive cartilage degeneration, subchondral bone remodeling, osteophyte formation, and synovial inflammation (2). The advance of these injuries will lead to maladaptive repair responses, which will exacerbate the process as the body tries to make up for the damage.

Articular cartilage is a specialized avascular connective tissue composed primarily of type II collagen and proteoglycans, with chondrocytes as the only resident cell type. In OA, chondrocytes undergo some changes, becoming hypertrophic and metabolically active, and begin producing matrix-degrading enzymes such as matrix metalloproteinases (MMPs) and aggrecanases, as well as proinflammatory cytokines like interleukin-1β (IL-1β) and tumor necrosis factor-alpha (TNF-α). These biochemical alterations accelerate ECM breakdown and contribute to hallmark features of OA, including cartilage fibrillation, loss of proteoglycan content, chondrocyte clustering, and eventual erosion down to the subchondral bone (3). Due to the limited capacity to regenerate, the degeneration of articular cartilage plays a central role in the onset and progression of osteoarthritis. Preclinical research is therefore crucial for understanding the mechanisms governing its degeneration and assessing potential treatments that may maintain or restore its integrity.

The rabbit is one of the most widely used species in preclinical OA research, offering several practical advantages including cost-effectiveness, ease of handling and housing, and a knee joint of suitable size for surgical manipulation and histological analysis (4). Among experimental models, surgically induced OA via anterior cruciate ligament transection (ACLT) is a well-established method, inducing cartilage lesions on rabbits similar to those in humans (5).

Regarding the methods used in preclinical studies of OA, histological methods are the most commonly used, but the experience and subjectivity of the professional who is evaluating the samples can affect the results (6). To obtain qualitative and quantitative measurements of the structural changes in the articular cartilage and subchondral bone during the development of OA, there are histomorphometric analysis techniques that allow us to study these samples more objectively and accurately, achieving a reproducible method. However, the histomorphometric study also has its limitations, such as the requirement for specialized software and hardware (e.g., digitizing tablets or high-resolution monitors) to ensure accurate quantification (7).

In addition to structural assessment, histology and immunohistochemistry (IHC) provide complementary, valuable insight into the structural and molecular processes underlying OA. In preclinical and clinical studies of cartilage repair and degeneration, histological analysis serves as a fundamental tool for evaluating structural outcomes. It provides essential information on the composition, organization, and integrity of the extracellular matrix (ECM), as well as the cellular architecture in comparison with native cartilage (8). Moreover, by enabling the detection and localization of specific biomarkers within tissue sections, IHC allows researchers to evaluate the expression of proinflammatory cytokines, matrix-degrading enzymes, and structural proteins associated with cartilage metabolism. According to Hoemann et al. (8), a comprehensive histological endpoint analysis should integrate both conventional staining methods—such as Safranin O for proteoglycan content—and immunostaining for collagen types I and II, in order to accurately assess cartilage repair, subchondral bone integrity, and osteochondral integration.

Avocado/soybean unsaponifiables (ASU) are natural lipid extracts composed of phytosterols, beta-sitosterol, campesterol and stigmasterol. Phytosterols, are considered potent anti-inflammatory agents with antioxidant, analgesic and chondroprotective properties. *In vitro* studies have demonstrated that ASU can stimulate aggrecan synthesis and downregulate the expression of inflammatory mediators (IL-1β, TNF-α) and catabolic enzymes (MMPs) in chondrocytes (9–11). Complementarily, preclinical studies in various models have suggested beneficial structural effects, such as maintained matrix staining, decreased cellular abnormalities, partial preservation of cartilage integrity (12, 13). However, the efficacy of ASU remains inconsistent across different species and experimental conditions, and quantitative histomorphometric evidence in rabbit models is currently lacking. Given the need to further characterize the structural potential of ASU as a disease-modifying agent, this study aimed to quantitatively assess whether ASU may eventually function as disease-modifying agents. The therapeutic effect was evaluated through a combination of histomorphometric, histological, and immunohistochemical analyses, providing both structural and molecular insights into their potential for cartilage preservation.

## 2 Materials and methods

### 2.1 Experimental animal model

The study was carried out with 24 healthy male New Zealand White rabbits (Granja San Bernardo, Navarra, Spain) of 6–7 months of age with a mean body weight of approximately 5.0 kg. Data from previous experiments on rabbits with similar protocols were used to calculate the sample size (14). The fibrillation index differences between treated control and osteoarthritic groups were around 0.08 mm with a standard deviation of around 0.05 mm. With these differences, 8 animals per group were sufficient to reject the null hypothesis that the response difference was zero with a power of 0.8 and a significance level of 0.05 (Experimental Design Assistant, NC3Rs).^1^

The experimental protocol was approved by the Ethics Committee of the University of Santiago de Compostela (reference no. 01/16/LU-002) and *in vivo* procedures were performed in accordance with Spanish legislation on the protection of animals used for scientific purposes (Ley 14/2007, Real Decreto 53/2013 and its subsequent amendments) and European Union Directive 2010/63/EU on the protection of animals used for scientific purposes. Also, this article was drafted following the Animals in Research *In Vivo* Experiments (ARRIVE) guidelines (15).

Upon arrival at the animal facility of the University of Santiago de Compostela (Lugo, Spain), the animals were kept for a three-week acclimatization period. During this time and throughout the experiment, the rabbits were housed in individual enriched cages (R-suite, Techniplast, Varese, Italy) with autoclaved herbs, fresh fruit pieces, paper rolls, and wooden sticks as environmental enrichment. The light cycle was set at 12 h light/dark, with controlled temperature and humidity. Food and water were offered ad libitum. Finally, a veterinary team trained in the care and supervision of laboratory animals checked daily the general condition and health status of each rabbit.

After acclimatization, osteoarthritis (OA) was induced in 16 rabbits (8 per treatment group) by anterior cruciate ligament transection (ACLT) surgery plus partial meniscectomy (16) performed on the right knee in half of the animals and on the left knee in the other half, to generate instability and develop OA. The contralateral knee served as a healthy control. In the remaining 8 rabbits (SHAM group), an arthrotomy was performed to reproduce surgical manipulation without inducing instability.

For anesthetic premedication, animals received a combination of ketamine (25 mg/Kg IM, Imalgène 1000, Merial, Toulouse, France), medetomidine (50 μg/Kg IM, Domtor, Esteve, Barcelona, Spain) and buprenorphine (0.03 mg/Kg IM, Buprex, RB Pharmaceuticals, Berkshire, UK) to ensure adequate analgesia and sedation during the surgical procedure. Isoflurane was used as general anesthesia (Inspiratory Fraction ISO 2.5–4%, Isoflurane, Schering-Plow, Madrid, Spain) for maintenance, administered with the aid of a facemask. As antibiotic prophylaxis, enrofloxacin (5 mg/Kg SC Ganadexil 5%, Invesa, Barcelona, Spain), and for pain control, meloxicam (0.2 mg/Kg SC, Metacam, Boehringer Ingelheim España, Barcelona, Spain) was administered for 5 days.

Three weeks after the induction of OA, treatment was initiated and continued for 24 weeks. The SHAM group received oral placebo treatment (2 mL NaCl 0.9%). The CONT group received the same placebo treatment. The ASU group received a 50 mg/day (10 mg/kg/day) oral dose of ASU, adapted from the standard human clinical dose of 300 mg/day. This dosage was selected based on previous studies in human clinical trials and preclinical models using similar regimens (17, 18). Animals were randomly allocated to the SHAM, CONT, or ASU groups using a manual lottery-based method to ensure unbiased assignment. The personnel responsible for administering the treatments remained blinded to the group to which each animal belonged. During the treatment period, body weight was recorded weekly and general condition and signs of pain or discomfort were monitored daily.

At 27 weeks post-surgery (i.e., after 3 weeks of waiting and 24 weeks of treatment), euthanasia was performed. For this purpose, the animals were sedated with ketamine (25 mg/kg IM, Imalgène 1000, Merial, Toulouse, France) and medetomidine (50 μg/kg IM, Domtor, Esteve, Barcelona, Spain), and then sodium pentobarbital 100 mg/kg IV was administered into the lateral auricular vein (Dolethal, Vétoquinol, Madrid, Spain). After confirming death, the two knees of each rabbit were dissected, carefully removing the soft tissues surrounding the joint. The samples were fixed in 10% buffered formalin.

**Figure 1:**
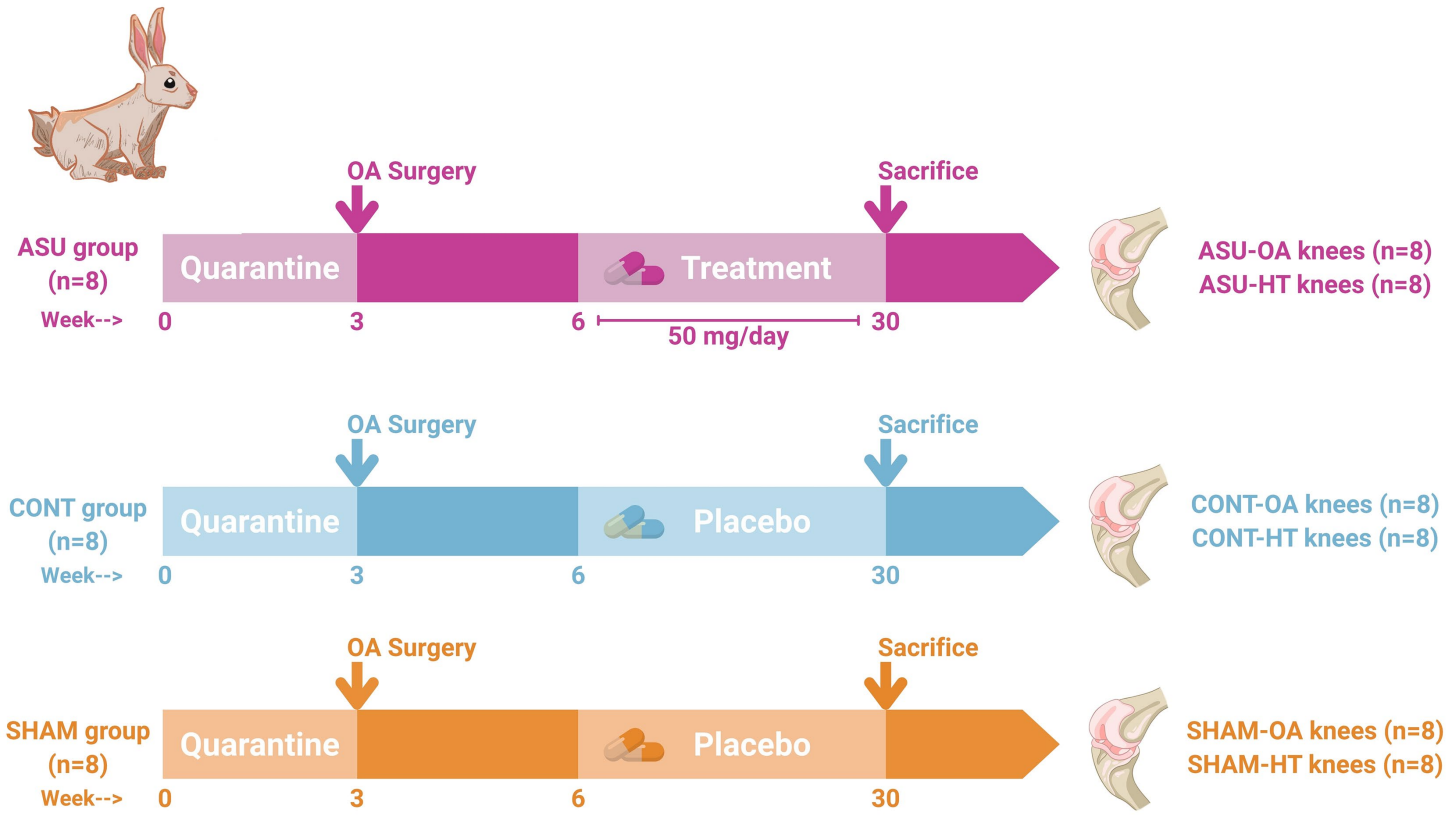
Experimental design. Rabbits were divided into three groups. Operated joints constituted OA groups and contralateral joints were used as healthy controls. ASU: Operated knees treated with avocado/soybean unsaponifiables; CONT: control, operated knees treated with placebo; SHAM: sham surgery, treated with placebo.

For specimen organization, each knee was classified into one of the following six groups: sham operated knees (SHAM-OA, *n* = 8), contralateral non-operated knees (SHAM-HT, *n* = 8), operated knees of control group (CONT-OA, *n* = 8), contralateral non-operated knees of control group (CONT-HT, *n* = 8), operated knees of ASU group (ASU-OA, *n* = 8) and non-operated contralateral knees of ASU group (ASU-HT, *n* = 8) (Figure 1).

### 2.2 Histological preparations

After extraction and fixation in 10% buffered formalin, standardized sections were obtained from each medial and lateral femoral condyle twice (MFC, LFC), as well as from the medial and lateral tibial plateaus (MTP, LTP), using a band saw. Half of the samples were processed without decalcification, underwent a dehydration process, and were embedded in glycolmethacrylate resin (Technovit 7200-VLC, Heraeus Kulzer GmbH, Werheim, Germany) following the method standardized by Donath (19). Subsequently, the sections were cut and polished using a controlled grinding machine (EXAKT Apparatebau, Norderstedt, Germany) until a final thickness of approximately 65 μm. The slices obtained were stained using the Lévai-Laczkó histological technique (20).

The other half of the samples were decalcified (Osteodec, Bio-Optica, Milano, Italy), dehydrated, and embedded in paraffin. They were then sectioned of 5 μm in thickness using a microtome (Leica RM 2255, Leica Biosystems Nusshoch GmbH, Germany), some sections were stained with Hematoxylin–Eosin (HE) for histological evaluation, and others were processed for immunohistochemical analyses.

Decalcified and non-decalcified histological sections were digitized using a Leica motorized light microscope (Leica DM6 B, Leica Microsystems, Germany) equipped with a digital camera and connected to a PC-based image capture system (Leica LAS X, Leica Microsystems, Germany). Images were obtained at magnifications up to ×4 and ×10.

### 2.3 Histomorphometric analysis

Histomorphometric evaluation of the non-decalcified sections was performed following the methodological recommendations for the analysis of cartilage and subchondral bone, in animal models of osteoarthritis, described by Pastoureau et al. (21). Two PC-based image analysis software programs: Image-Pro Premier 11.1 (Media Cybernetics, Bethesda, MD, USA) for the articular cartilage measurements and CellSens 4.4.1 (Olympus Corporation, Japan) for the trabecular bone analysis.

All samples were analyzed in random order and evaluated independently by two blinded observers, according to the Osteoarthritis Research Society International (OARSI) criteria for histomorphometric assessment (5).

**Figure 2:**
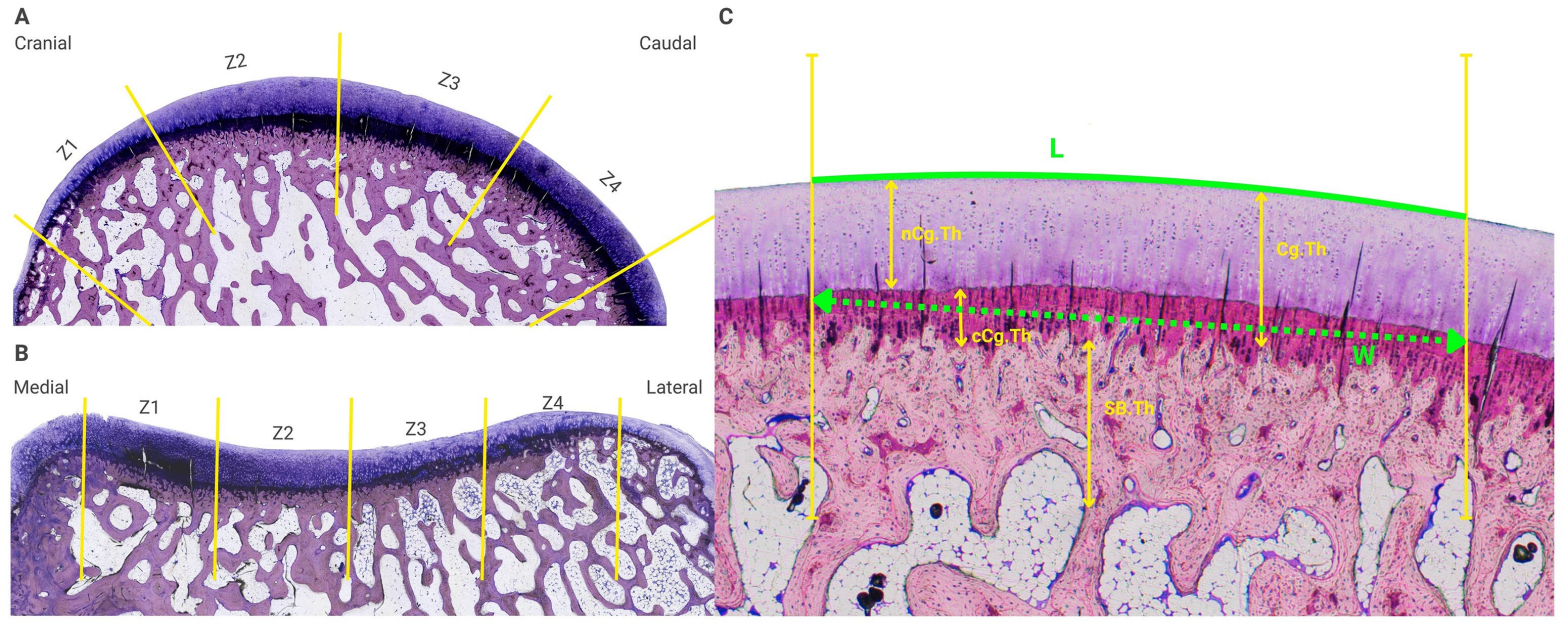
Histomorphometric analysis. **(A)** Femoral condyle ROI; **(B)** Tibial plateau ROI; **(C)** Cartilage and subchondral bone thickness: nCg.Th, non-calcified cartilage thickness; cCg.Th, calcified-cartilage thickness; Cg.Th, total cartilage thickness; Sb.Th, subchondral bone cortical thickness. Fibrillation index (FI) calculated as the quotient between the length of the cartilage surface (L) and a straight parallel with the same width line (W).

For each section, a region of interest (ROI) was established delimiting the articular cartilage margins. This ROI was divided into four zones of equal distance in the anteroposterior direction (Figures 2A,B). In each of these zones, cartilage and subchondral bone thickness measurements were taken and the following morphometric variables were calculated (Figure 2C):

- Non-calcified cartilage thickness (nCg.Th, μm): the mean distance measured from the articular surface to the tidemark line.
- Calcified cartilage thickness (cCg.Th, μm): the mean distance measured from the tidemark to the subchondral bone plate.
- Total cartilage thickness (Cg.Th, μm): resulting from the sum of nCg.Th and cCg.Th.
- Subchondral bone cortical thickness (Sb.Th, μm): the mean distance measured from the subchondral bone plate to the beginning of the trabecular bone.

In addition, the Fibrillation Index (FI) was calculated as an indicator of the degree of superficial fissures and undulations. The FI was determined by performing the quotient between the actual length of the cartilage surface (L) and the length of a parallel straight line of the same width (W) at the height of the tide mark, which served as a reference to an ideally flat surface (7) (Figure 2C).

**Figure 3:**
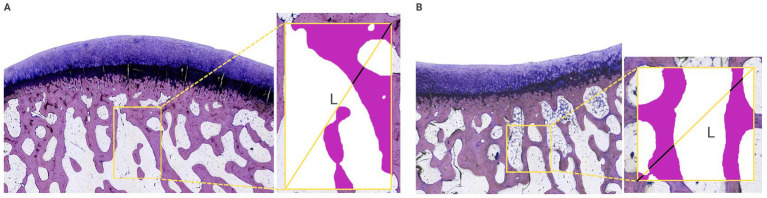
Trabecular subchondral bone histology. **(A)** Femoral ROI (1.5 × 2.5 mm); **(B)** Tibial ROI (1.5 × 1.5 mm). The yellow rectangles indicate the ROIs. The yellow diagonal line (L) indicates the reference axis used for trabecular measurements, and the black segments along L denote the portions used to assess trabecular thickness (Tb.Th) and trabecular number (Tb.N). Segmented trabecular bone within the ROIs is shown in magenta.

For the analysis of trabecular bone (Figure 3), specific ROIs were defined immediately below the articular cartilage, centered on the medial axis of each specimen. The ROI size was 2.5 × 1.5 mm for femoral specimens and 1.5 × 1.5 mm for tibial specimens. The diagonal line (L, mm) crossing each ROI was used for linear measurements of trabecular architecture; a femoral line (L_F_) and a tibial line (L_T_) were defined for the respective ROIs. The nomenclature of structural parameters of trabecular bone was used according to the American Society for Bone and Mineral Research (ASBMR) guidelines (22). Histomorphometric parameters evaluated in trabecular bone in both femoral condyles and tibial plateaus were:

- Trabecular area (Tb.A, %): the percentage of segmented trabecular tissue relative to the total ROI area.
- Trabecular thickness (Tb.Th, mm): mean trabecular thickness of the trabeculae intersected by L.
- Trabecular number (Tb.N, 1/mm): number of trabeculae intersected by L per unit length of L.
- Trabecular separation (Tb.Sp, mm): obtained from the following formula: Tb.Sp = (1/Tb.N)-Tb.Th.

### 2.4 Microscopical evaluation

Cartilage changes were evaluated in the decalcified sections by two independent observers. The histological scoring system was adapted from the table published by Fernández-Martín et al. (23). The parameters evaluated were:

- Severity of cartilage pathology
- Severity of chondrocyte pathology
- Severity of proteoglycan pathology
- Tidemark integrity

The cartilage, chondrocyte, and proteoglycan pathology in these decalcified samples ranged from 0 (normal) to 4 (totally afflicted), while the tidemark ranged from 0 to 2 (Table 1).

**Table 1:** Scoring system for histological evaluation of articular cartilage pathology.

Cartilage	Score
Severity of cartilage pathology characteristics	
Normal volume, smooth surface with all zones intact	0
Surface undulations including fissures in surface/upper zone	1
Fissures to mid zone and/or erosion of surface/upper zone	2
Fissures to deep zone and/or erosion through mid zone	3
Full thickness loss of cartilage	4
Severity of chondrocyte pathology characteristics	
Normal	0
Loss of superficial cells or relative increased density with occasional clusters	1
Small clusters (2–4 cells) predominate	2
Large clusters (≥5 ells) predominate	3
Cell loss (necrosis/apoptosis) predominate	4
Tidemark	
Intact and distinct	0
No consistent or distinct (loss and/or duplication)	1
Loss of tidemark which is crossed by blood vessels	2
Total	10

Scores reflect severity of cartilage lesions, chondrocyte pathology, and tidemark changes.

### 2.5 Immunohistochemical evaluation

Decalcified sections were deparaffinized in xylene, rehydrated through a graded ethanol series, and subjected to antigen retrieval via enzymatic digestion with proteinase K. After three washes in Phosphate-Buffered Saline (PBS), endogenous peroxidase activity was blocked (BLOXALL® Endogenous Blocking Solution, Vector Laboratories, SP-6000, Burlingame, CA, USA), and nonspecific binding was blocked with normal horse serum (Normal Horse Serum Blocking Solution, 2.5%, Vector Laboratories, S-2012, Burlingame, CA, USA).

Primary antibodies against mouse monoclonal anti-collagen type II antibody (Clone 5B2.5, dilution 1:100, Novus Biologicals, NB600-844, Littleton, CO, USA) and rabbit polyclonal anti-collagen type X antibody (dilution 1:200, Cusabio, CSB-PA005715ESR1HU, Wuhan, China) were incubated in a humid chamber for 12 h at 4 °C. Sections were then washed in PBS-Tween 20 and incubated with biotinylated goat anti-mouse IgG (H + L) secondary antibody (dilution 1:200, Vector Laboratories, BA-9200, Burlingame, CA, USA) for 30 min.

Detection was performed with diaminobenzidine using a commercial kit (ImmPACT® DAB Peroxidase Substrate Kit, Vector Laboratories, SK-4105, Burlingame, CA, USA), and sections were counterstained with HE. Positive and negative controls were included in each staining batch.

**Figure 4:**
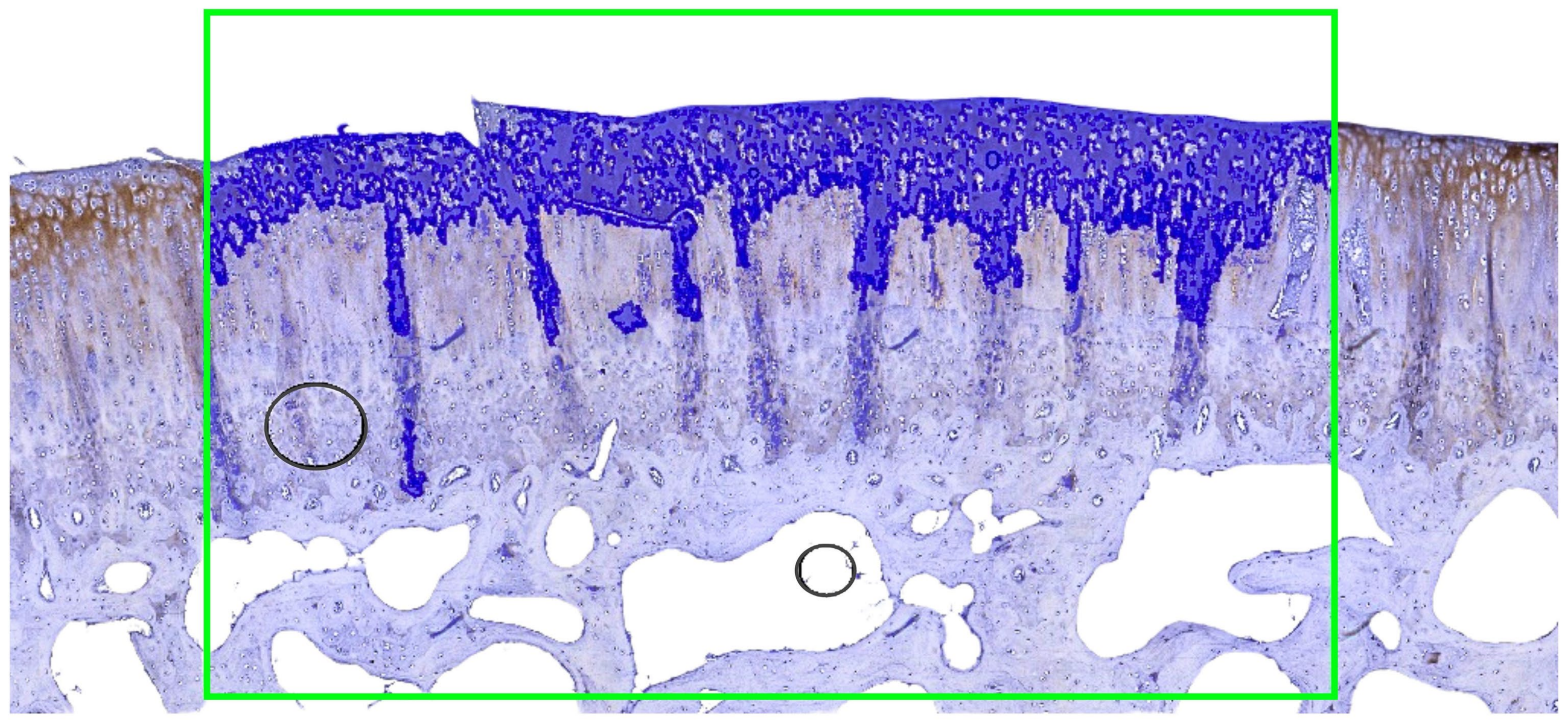
Immunohistochemical positivity quantitatively analyzed using Image-Pro Premier software. Representative regions of interest (ROIs, 2.5 × 1.5 mm) selected at the interface between zones 2 and 3. The green area indicates the ROI, the blue areas indicate positive staining, and the black circles indicate background selection for thresholding and quantification.

Immunohistochemical positivity was quantitatively analyzed using Image-Pro Premier software version 11.1 (Media Cybernetics, Bethesda, MD, USA). Regions of interest (ROIs) similar to those used for the histomorphometric analysis (2.5 × 1.5 mm) were selected at locations approximately corresponding to the boundary between zones 2 and 3, as previously defined in the histomorphometric assessment. The Count Smart tool was applied by manually defining representative areas of positive staining and background, allowing the software to automatically calculate the percentage of positive areas within each ROI. This approach provided an objective and reproducible quantification of antigen expression levels (Figure 4).

### 2.6 Statistical analysis

Data were expressed as means ± standard deviations (SDs). Statistical analysis was performed using SigmaPlot software version 12.5 (Systat Software Inc., Chicago, IL, USA). Student’s t-test was used in those cases where data followed a normal distribution for comparisons between two groups HT vs. OA, e.g., CONT OA vs. CONT HT. When the assumption of normality was not found, the nonparametric Mann–Whitney U test was applied.

For comparison between all groups, a one-way ANOVA was used, followed by the Holm-Sidak *post hoc* test. In cases where the data were not normally distributed, the Kruskal-Wallis nonparametric test was used, followed by post hoc analysis using Dunn’s test.

For immunohistochemical analysis, comparisons of positivity between groups, for example SHAM versus CONT, were conducted using Student’s t-test when data met the assumption of normality. When this assumption was violated, the nonparametric Mann–Whitney U test was applied, following the same procedure used in the histomorphometric analysis.

A value of *p*-value of less than 0.05 was considered statistically significant.

## 3 Results

The procedures were carried out without any problems, and the treatments were well accepted. Neither weight nor general condition changed during the procedure. For unclear circumstances, only one rabbit died during the surgery.

### 3.1 Histomorphometric results

**Figure 5:**
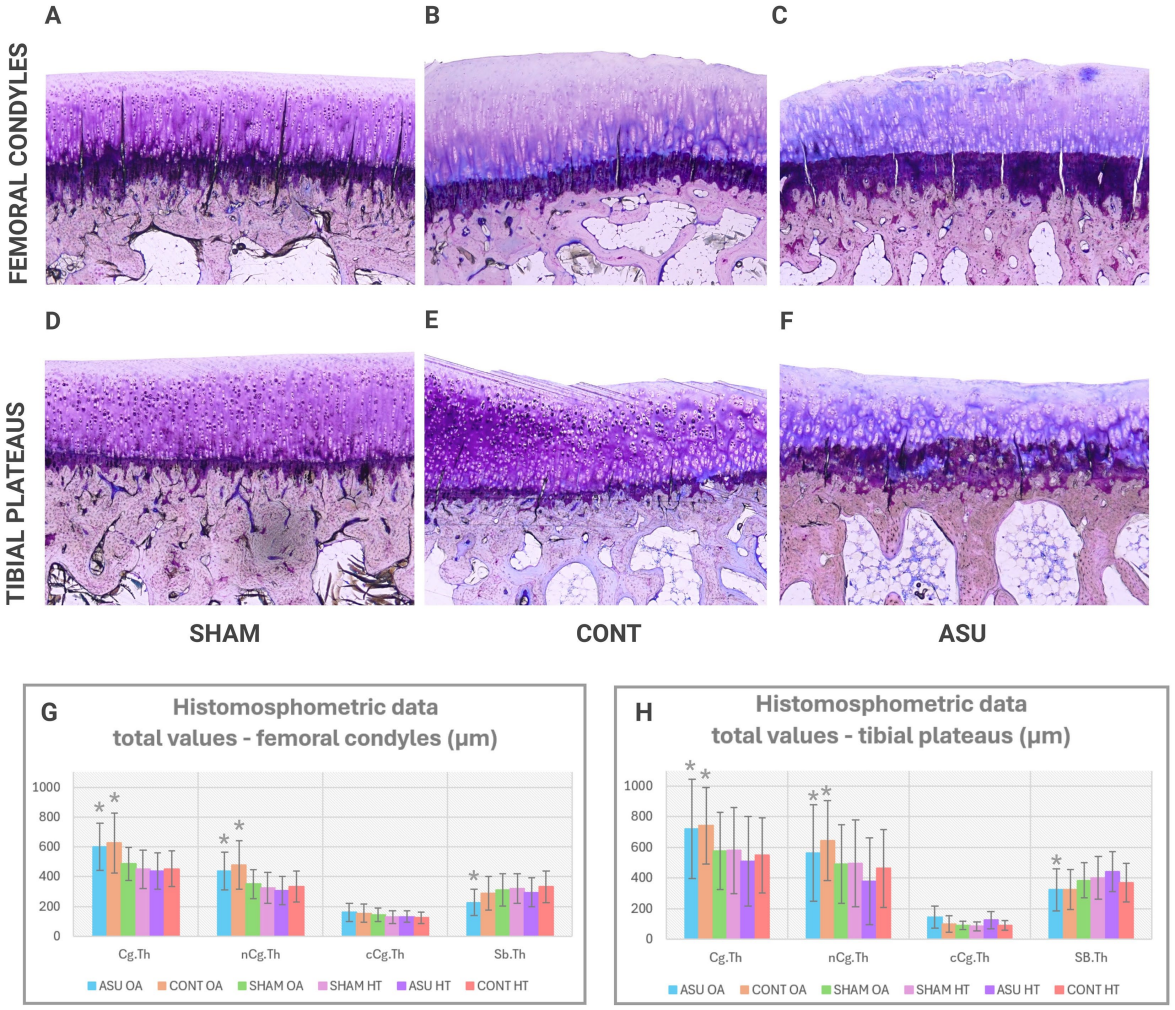
Articular cartilage morphology and histomorphometry in OA femoral condyles and tibial plateaus. Representative Lévai-Laczkó-stained sections from **(A,D)** SHAM: sham surgery, treated with placebo; **(B,E)** CONT: control, operated knees treated with placebo; **(C,F)** ASU: operated knees treated with avocado/soybean unsaponifiables. **(G,H)** Histomorphometric measurements of total cartilage thickness (Cg.Th), non-calcified cartilage thickness (nCg.Th), calcified cartilage thickness (cCg.Th), and subchondral bone plate thickness (Sb.Th) across all groups. Asterisks (*) indicate significant differences with their contralateral HT joint.

The data obtained from the histomorphometric analysis of the femoral condyles and tibial plateaus of the articular cartilage and subchondral bone are shown in Figure 5 and Table 2. Total values correspond to the mean of the eight zonal measurements obtained from the four zones of the medial and lateral compartments (Supplementary Tables S1, S2).

**Table 2:** Histomorphometric data from the analysis of femoral condyles and tibia plateaus.

Cartilage compartments	Study groups		Femoral condyles	Total	Lateral				Medial				Tibial plateaus	Total	Lateral				Medial			
					Z1	Z2	Z3	Z4	Z1	Z2	Z3	Z4			Z1	Z2	Z3	Z4	Z1	Z2	Z3	Z4
Cg.Th	OA	SHAM vs. CONT		✓			✓	✓						✓			✓					
		SHAM vs. ASU		✓			✓							✓			✓					
		CONT vs. ASU																				
	OA vs HT	SHAM-OA VS HT																				
		CONT-OA VS HT		✓	✓		✓	✓						✓		✓	✓					
		ASU-OA VS HT		✓			✓							✓		✓	✓					
nCg.Th	OA	SHAM vs. CONT		✓			✓	✓						✓			✓					
		SHAM vs. ASU		✓			✓															
		CONT vs. ASU						✓														
	OA vs HT	SHAM-OA VS HT																				
		CONT-OA VS HT		✓			✓	✓						✓			✓	✓				
		ASU-OA VS HT		✓			✓							✓								
cCg.Th	OA	SHAM vs. CONT					✓															
		SHAM vs. ASU					✓							✓		✓						
		CONT vs. ASU										✓		✓		✓						
	OA vs HT	SHAM-OA VS HT																				
		CONT-OA VS HT																				
		ASU-OA VS HT														✓						
Sb.Th	OA	SHAM vs. CONT		✓																		
		SHAM vs. ASU			✓																	
		CONT vs. ASU																				
	OA vs HT	SHAM-OA VS HT																				
		CONT-OA VS HT		✓																		
		ASU-OA VS HT		✓										✓								

Significant differences between study groups are indicated by ✓. Empty cells indicate non-significant differences. Comparisons were performed within osteoarthritic groups (OA), as well as between OA and their contralateral healthy (HT) joint, at each anatomical region, side, and zone. Total cartilage thickness (Cg.Th), non-calcified cartilage thickness (nCg.Th), calcified cartilage thickness (cCg.Th) and subchondral bone plate thickness (Sb.Th). Zones 1 to 4 (Z1–Z4).

In the analysis of articular cartilage, the total cartilage thickness (Cg.Th) showed a significant increase in both anatomical regions in the ASU-OA and CONT-OA groups compared with their respective HT groups (indicated by asterisks *) (Figures 5G,H) and with the SHAM-OA group. At the zonal level, significant differences were observed in Z3 in both anatomical regions between SHAM-OA and the ASU-OA and CONT-OA groups, while in Z4 the only significant difference was found in the lateral femoral condyle between SHAM-OA and CONT-OA.

For non-calcified cartilage thickness (nCg.Th), significant differences in the mean values were observed in the femoral condyles between ASU-OA and CONT-OA and their respective HT groups, as well as between both OA groups and SHAM-OA. At the zonal level, the lateral femoral condyle showed no differences in cranial zones (Z1 or Z2), whereas Z3 showed significant differences between CONT-OA and ASU-OA with SHAM-OA and their contralateral HT group. In Z4, differences were observed between ASU-OA and both groups CONT-OA and SHAM-OA, and between CONT-OA and its contralateral HT group.

In the tibial plateaus, significant differences in the mean values were observed between ASU-OA and CONT-OA with their respective HT groups, as well as between CONT-OA and SHAM-OA. Zonal analysis of the lateral tibial plateau showed no differences in medial zones (Z1 or Z2), whereas Z3 differed between CONT-OA and SHAM-OA. Lateral zones (Z3 and Z4) showed differences between CONT-OA and its contralateral HT group.

For calcified cartilage thickness (cCg.Th), no significant differences were found in the mean values of the femoral condyles. However, zonal analysis of the medial femoral condyle revealed differences in Z3 between ASU-OA and CONT-OA with SHAM-OA. In the tibial plateaus, significant differences were observed in the mean values between ASU-OA and CONT-OA and between ASU-OA and SHAM-OA. At the zonal level, the lateral tibial plateau showed differences in Z2 between ASU-OA and CONT-OA, between ASU-OA and SHAM-OA, and between ASU-OA and its contralateral HT group.

Regarding the subchondral bone (Sb.Th), significant differences were observed in mean values between ASU-OA and ASU-HT in both anatomic regions (Figures 5G,H). In lateral femoral condyles, significant differences in mean values were also observed between SHAM-OA and CONT-OA, whereas no significant differences were detected between CONT-OA and ASU-OA. At the zonal level, no significant differences were found in either the medial or lateral compartments of the femoral condyles or tibial plateaus, except for ASU-OA versus SHAM-OA in Z1 of the lateral femoral condyle.

**Figure 6:**
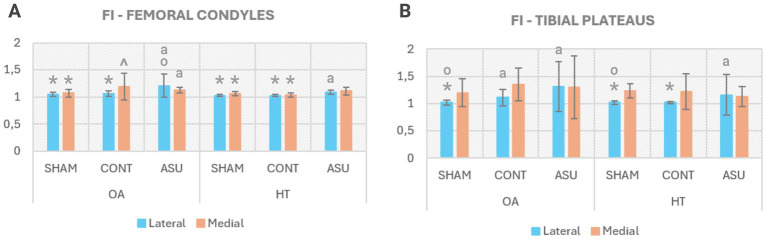
Fibrillation index. **(A)** Femoral condyles; **(B)** Tibial plateaus. Bars represent mean ± SD of the lateral and medial compartments. Statistical differences are indicated as follows: ª vs. SHAM OA, ° vs. CONT OA, * vs. ASU OA, and ^ vs. contralateral healthy (HT) joint.

The fibrillation index results (Figure 6; Supplementary Table S3) showed significant differences in the lateral femoral condyles between SHAM-OA and ASU-OA, and between CONT-OA and ASU-OA. In the medial femoral condyle, significant differences were found between SHAM-OA and ASU-OA, as well as between CONT-OA and its contralateral HT group. In the lateral tibial plateau, SHAM-OA differed significantly from both CONT-OA and ASU-OA, whereas no significant differences were detected in the medial tibial plateau.

In the analysis of trabecular bone (Supplementary Table S4), most parameters showed no significant differences between groups. For trabecular area (Tb.A), a significant difference was observed in the lateral tibial plateau between SHAM-OA and ASU-OA. For trabecular number (Tb.N), a significant difference was detected in the medial tibial plateau between CONT-OA and the contralateral HT group. No significant differences were found for trabecular thickness (Tb.Th) or trabecular separation (Tb.Sp) in either femoral condyles or tibial plateaus.

In summary, the statistically significant differences were primarily observed in caudal zones of lateral femoral condyles and in the central zones (Z2–Z3) of the lateral tibial plateaus, particularly for Cg.Th and nCg.Th, between the OA groups (ASU-OA, CONT-OA) and their respective controls (SHAM-OA, HT). These findings suggest a pattern consistent with the primary load-bearing regions of both articular surfaces (especially Z3 in lateral femoral condyles), accompanied by some changes in Sb.Th, fibrillation index, and minimal trabecular alterations.

### 3.2 Microscopical evaluation

**Figure 7:**
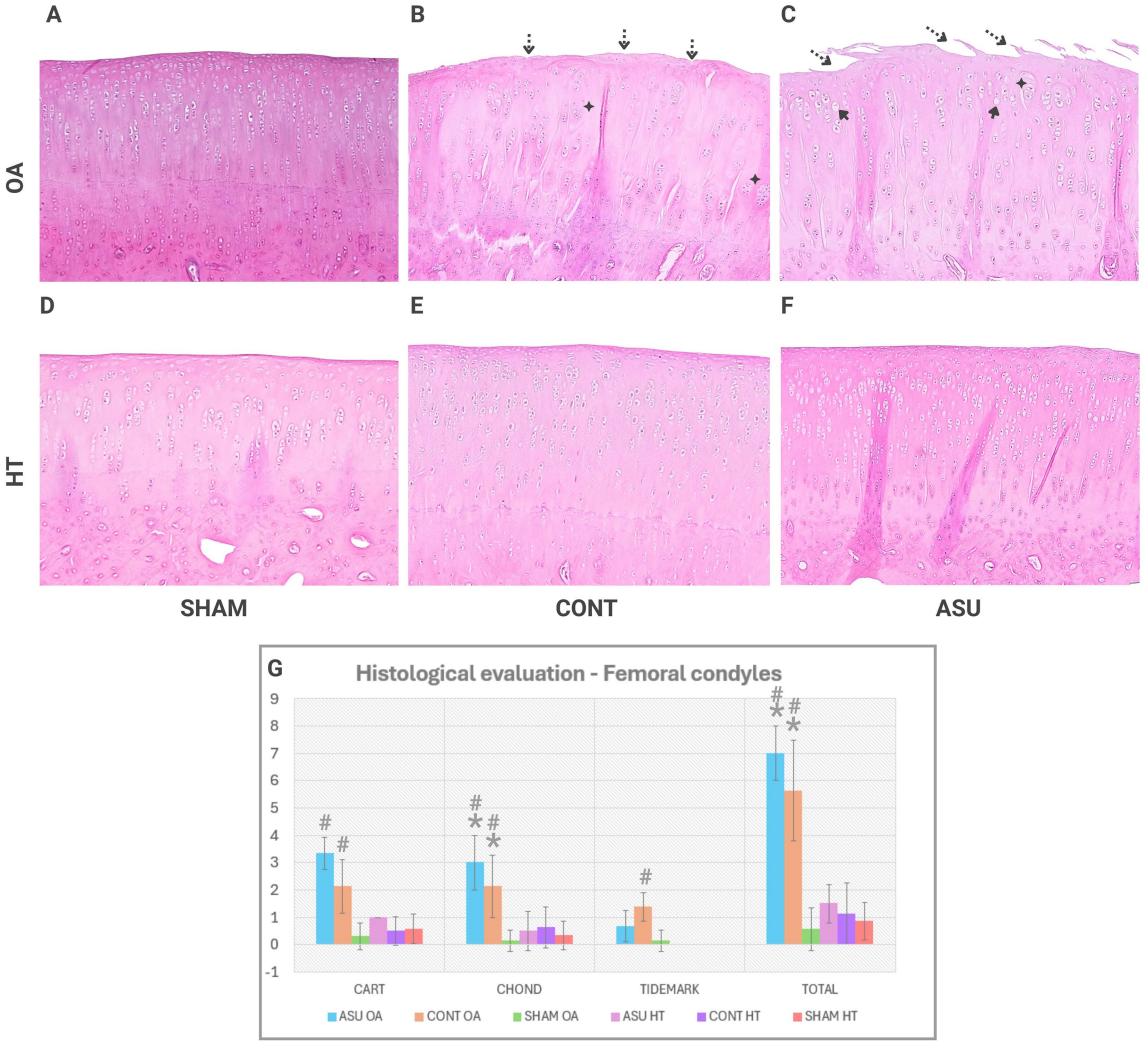
Histological cartilage evaluation in femoral condyles. Representative hematoxylin–eosin-stained sections of osteaoarthritic (OA) femoral condyles and their contralateral healthy (HT) from **(A,D)** SHAM: sham surgery, treated with placebo; **(B,E)** CONT: control, operated knees treated with placebo; **(C,F)** ASU: operated knees treated with avocado/soybean unsaponifiables. **(G)** The bar graph shows semiquantitative histological scores for cartilage structure (CART), chondrocyte abnormalities (CHOND), tidemark integrity (TIDEMARK), and total score (TOTAL) in the previously mentioned groups. Asterisks (*) indicate significant differences with the contralateral HT joint and the hash (#) indicates significant differences with the SHAM-OA group. Four-point stars denote chondrocyte clusters, black arrows denote empty lacunae, and dashed arrows denote surface undulations and irregularities.

Histological evaluation for cartilage, chondrocyte and tidemark integrity, are shown in Figure 7. Microscopic evaluation revealed characteristic degenerative changes in ASU-OA (Figure 7C) and CONT-OA groups (Figure 7B), including surface undulations and fissures extending into superficial and middle cartilage zones, chondrocyte clustering (>5 cells per lacuna), and occasional areas of cell loss. In contrast, SHAM-OA and contralateral healthy joints (HT) (Figure 7A) maintained smoother articular surfaces with regular chondrocyte alignment and preserved matrix organization.

Cartilage pathology scores were significantly higher in ASU-OA and CONT-OA compared with SHAM-OA (*p* < 0.05), whereas no significant differences were detected between ASU-OA and CONT-OA. Chondrocyte pathology showed a similar pattern, with significantly higher scores in ASU-OA and CONT-OA than in SHAM-OA, and no significant differences between ASU-OA and CONT-OA. In addition, OA groups (ASU-OA and CONT-OA) presented significantly higher chondrocyte scores than their respective healthy contralateral knees (HT) (Figure 7D), while no significant differences were observed between HT groups and SHAM-OA. For tidemark integrity, significant differences were found between CONT-OA and both SHAM-HT and SHAM-OA, whereas no significant differences were observed between CONT-OA and ASU-OA, or between ASU-OA and SHAM-OA.

The total histological score followed the same general pattern as the individual parameters. ASU-OA and CONT-OA showed significantly higher total scores than SHAM-OA, with no significant differences between ASU-OA and CONT-OA. Furthermore, total scores were significantly higher in ASU-OA and CONT-OA groups than in HT knees (Figure 7D), and no significant differences were found between HT groups and SHAM-OA.

### 3.3 Immunohistochemical results

**Figure 8:**
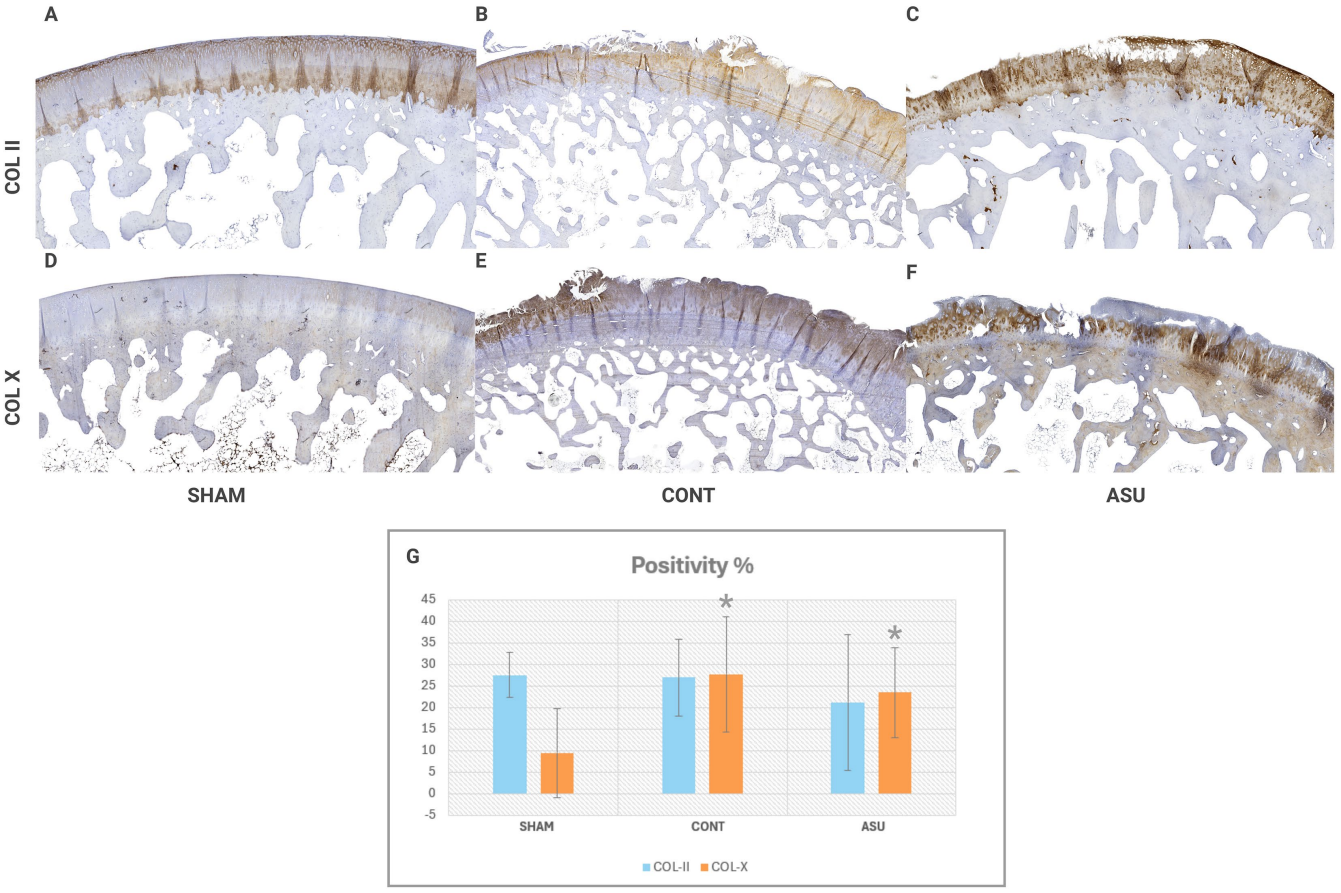
Immunohistochemical positivity for Col-II and Col-X of the three study groups. **(A,D)** SHAM: sham surgery, treated with placebo; **(B,E)** CONT: control, operated knees treated with placebo; **(C,F)** ASU: operated knees treated with avocado/soybean unsaponifiables; **(G)** Positivity percentage of collagen type-II and type-X. Asterisks (*) indicate significant differences with the SHAM group.

The immunohistochemistry showed (Figures 8A–F) a trend toward increased Col-II positivity was observed in the SHAM group compared to Col-X, which showed minimal positivity. In contrast, the CONT and ASU groups exhibited an opposite trend, with Col-II positivity being slightly lower than that of Col-X.

The statistical analysis of both collagens, summarized in Figure 8G, revealed no significant differences in type II collagen (Col-II) positivity among the groups. Conversely, type X collagen (Col-X) exhibited significant differences between the SHAM and CONT groups, and between SHAM and ASU groups, but not between CONT and ASU groups.

## 4 Discussion

This study investigated the long-term effects of ASU treatment in an experimental rabbit model of knee osteoarthritis through a comprehensive quantitative and qualitative analysis of both cartilage and subchondral bone. This approach enabled the evaluation of the potential therapeutic impact of ASU as a disease-modifying agent on cartilage and subchondral bone structures.

Surgically induced osteoarthritis (OA) models offer rapid onset, high reproducibility, and relatively low cost, yet they typically produce more severe lesions, faster structural progression, and attenuated therapeutic responsiveness compared with spontaneous or age-related models; these trade-offs should be explicitly considered during experimental design (24). In rabbits, the knee OA model is extensively characterized, with cartilage degeneration often detectable within 8–12 weeks post-induction and a predilection for the medial femoral condyle in many reports, paralleling patterns observed in humans and small-animal models (25). Nevertheless, interspecies differences in locomotion and joint anatomy can modify local load distribution and lesion topography: unlike bipeds, quadrupeds redistribute body weight across four limbs (26, 27), and rabbits also exhibit thinner articular cartilage and distinct zonal architecture relative to humans. All of which can complicate cross-species comparisons and may account for discrepancies such as the absence of significant medial-compartment differences in the present study (28). To disentangle procedure-related effects from disease-specific changes, inclusion of a sham surgery group was prioritized to control for factors such as altered weight bearing on the contralateral limb. In this cohort, no significant differences were detected between sham and healthy controls, supporting the interpretation that the observed changes reflect OA-specific pathology rather than nonspecific surgical or handling effects. However, contrary to these findings, McCoy et al. (29) describes that sham-operated animals can exhibit a certain degree of articular damage, likely related to surgical manipulation or altered joint mechanics, indicating that sham groups may occasionally show significant differences compared to healthy controls in some preclinical OA models (29).

In the present study, articular cartilage thickness was significantly increased in the OA groups relative to their corresponding healthy contralateral joints and to the sham-operated group. This increase was also reflected in the zonal analysis, where significant differences were observed in Z3 in both anatomical regions, and in Z4 only in the lateral femoral condyle. Overall, these findings are consistent with reports describing cartilage swelling or hypertrophy in OA, which may reflect a reparative or mechanoadaptive response to altered loading conditions. For instance, Fernández-Martín et al. (30) and Roemhildt et al. (31) described increased cartilage thickness primarily in medial load-bearing areas in osteoarthritic animal models, supporting the concept of regional cartilage remodeling linked to mechanical stress. However, cartilage thickness changes in OA are not uniform. Studies such as Eckstein et al. (32) and Sekiya et al. (33) have demonstrated heterogeneity in cartilage thickness changes during OA progression, including both thickening and thinning within different subregions of the same joint. These reports underscore that cartilage thickness alterations in OA are complex and stage-dependent, influenced by factors such as disease severity, anatomical location, species differences, and measurement methodologies.

Our histomorphometric analysis also revealed a significant decrease in subchondral bone thickness in OA groups compared with healthy controls, with the decrease being more pronounced in the tibial plateaus. Medial plateaus tended to show higher values than lateral regions. These results align with reports describing early subchondral bone remodeling in OA. As reviewed by Zhu et al. (34), subchondral bone undergoes aberrant remodeling in OA, with increased bone resorption and turnover preceding cartilage degeneration. Aho et al. (35) further corroborate that early-stage OA may manifest with subchondral plate thinning accompanied by elevated bone remodeling, particularly in medial compartments due to mechanical load redistribution. However, subchondral bone responses in OA are heterogeneous and evolve over disease progression. Bellido et al. (36) describe variants where in early stages of OA, there is a remodeling and resorption of the subchondral bone, but as OA progress, subchondral sclerosis and bone thickening predominate complicating a unified interpretation of bone changes. Thus, while our findings of reduced subchondral bone thickness in OA groups and relative medial thickening are consistent with early remodeling phenomena, the temporal and spatial heterogeneity of subchondral bone alterations must be considered when comparing across models and disease stages.

For cartilage integrity, both the cartilage thickness and fibrillation index (FI) analysis revealed significant differences between OA and HT groups, as well as between SHAM-OA versus the OA groups, while no significant differences were observed between CONT-OA and ASU-OA groups. These findings indicate that the surgical induction of OA produced a genuine osteoarthritic phenotype, as reflected by the differences between HT and SHAM-OA with OA groups, thereby validating the use of ACLT in rabbits as a suitable experimental model. This aligns with studies such as Yoshioka et al. (25), McCoy et al. (29) and Laverty et al. (5) which support the validity of this model for OA studies in rabbits. Conversely, other studies report significant differences between sham-operated and healthy controls, suggesting that the sham surgical procedure itself can induce mild cartilage alterations and inflammation, which may be misinterpreted about treatment efficacy and disease severity (37).

The absence of significant differences between ASU-OA and CONT-OA groups suggests a lack of measurable therapeutic effect of ASU treatment on the structural parameters assessed. This finding should be interpreted with caution, considering several limitations. Histomorphometry, while providing quantitative measurements, is highly dependent on operator expertise and lacks standardized protocols for image processing and parameter quantification (21, 38), which may have contributed to the variability observed in our measurements and the lack of significant differences between groups in some parameters. Similarly, in an equine surgically induced OA model, Kawcak et al. (39) did not demonstrate significant improvements in clinical signs with ASU treatment, although there was some reduction in cartilage erosion severity and synovial hemorrhage. This lack of effect on clinical signs mirrors our own results, where ASU treatment failed to produce measurable changes in structural parameters. In contrast, some other authors like Cake et al. (12), Boileau et al. (18) and de Paula et al. (40) found that ASU treatment confers a protective effect on articular cartilage morphology and attenuates subchondral bone sclerosis in surgical models.

No significant differences were found in trabecular bone parameters across most comparisons in our study. This limited response may reflect the disease stage analyzed or species-specific bone remodeling dynamics, as trabecular bone changes are known to vary according to OA progression and anatomical location (35). The few isolated differences detected in Tb.A and Tb.N suggest that trabecular involvement was modest compared with the more marked alterations observed in cartilage and subchondral plate thickness.

Regarding histological scoring, both cartilage and chondrocyte pathology, as well as total histological scores, were significantly higher in OA-operated knees (CONT-OA and ASU-OA) than in SHAM-OA and healthy contralateral joints, while no significant differences were detected between CONT-OA and ASU-OA. In relation to chondrocyte results, these findings indicate clear cellular alterations associated with OA induction, independent of ASU treatment. Conventional histological techniques such as HE and SO staining, while fundamental for assessing tissue architecture, suffer from subjectivity influenced by observer experience even when standardized scoring systems are used (28), which may have affected our ability to detect subtle treatment differences between CONT-OA and ASU-OA groups.

For tidemark integrity, significant differences were observed between CONT-OA and both SHAM-HT and SHAM-OA groups, whereas no significant differences appeared between CONT-OA and ASU-OA, or between ASU-OA and SHAM-OA. This irregular pattern aligns with the high variability in tidemark integrity reported by Fernández-Martín et al. (23) in rabbit OA models, who identified it as the most variable histological parameter.

Altogether, these results support successful OA induction and suggest that long-term ASU treatment may not have prevented the histological progression of joint degeneration. This interpretation contrasts with the protective effects reported by Cake et al. (12), who demonstrated relative preservation of cartilage architecture following ASU treatment in an ovine meniscectomy model, and by Boileau et al. (18), who found structural benefits in a canine experimental OA model. However, our findings align with Kawcak et al. (39), who reported no clinical improvements with ASU in equine OA despite some microscopic benefits, highlighting the inconsistent structural efficacy of ASU across different large-animal species and models.

The analysis of Col-II expression showed no significant differences among CONT, SHAM, and ASU groups but a tendency for greater Col-II positivity in the SHAM group, which contrasts with several published reports. Zhou et al. (41) described sustained Col-II positivity between weeks 2–4 followed by a decline after week 8. Similarly, Shi et al. (42) and Fang et al. (43) reported decreases in Col-II expression over time, while Meng et al. (44) observed significantly higher Col-II levels in non-OA groups compared to OA models at 16 weeks. These studies, performed in rabbit OA models, show a pattern of progressive loss of Col-II with OA development, which diverges from our observed statistical results.

One possible explanation for this discrepancy, especially given the long 27-week progression in our study, is that compensatory mechanisms may maintain Col-II expression despite cartilage damage. Studies have demonstrated that even in late-stage OA, chondrocytes can maintain or upregulate Col-II synthesis as an attempted repair or remodeling response (45). This dynamic balance between collagen degradation and synthesis may mask clear differences in Col-II levels despite ongoing pathology.

Regarding Col-X, we found significantly lower expression in the SHAM group compared to CONT and ASU, but no difference between CONT and ASU groups. This finding disagrees with Shirai et al. (46) in rabbits, who reported no significant differences in Col-X expression between OA, SHAM, and CONT groups. However, when compared to rat OA literature, our Col-X results align better with studies like Ping et al. (47) and Yan et al. (48), both documenting significant Col-X increases in OA groups versus sham controls, which might reflect species-specific responses or model variations.

Taken together, our results suggest that while the OA model was successfully implemented and Col-II and Col-X levels served as indicators of cartilage damage severity, ASU treatment did not appear to significantly modify markers of OA progression. The persistence of Col-II expression despite prolonged OA likely reflects a complex interplay of degenerative and reparative processes as well as spatial heterogeneity in collagen expression, highlighting the multifaceted dynamics of cartilage matrix remodeling in OA.

Overall, the observed cartilage thickening in OA groups may represent a reparative response or swelling secondary to early degeneration. Variations in subchondral bone thickness between medial and lateral compartments likely reflect biomechanical load redistribution typical of OA pathophysiology. The absence of significant differences between control and treated groups supports the limited therapeutic influence of ASU on cartilage and bone microstructure under these experimental conditions. However, we acknowledge that future studies including inflammatory marker analysis and functional outcomes (pain, mobility) would provide a more comprehensive assessment of ASU’s potential disease-modifying effects, particularly given its proposed anti-inflammatory mechanism of action.

In conclusion, this study reinforces the utility of surgically induced OA models for investigating early tissue alterations in osteoarthritis and evaluating potential disease-modifying therapies. While our findings contribute new quantitative data on the long-term effects of ASU treatment, several factors warrant critical reflection. Beyond the statistical power limitations associated with our sample size (*n* = 24 animals), the absence of a commercially available nutraceutical comparator and the focus on deep structural/molecular characterization, rather than functional assessments like range-of-motion or behavioral analysis, should be acknowledged. Furthermore, the 27-week longitudinal design, while robust for assessing chronic pathology, entails trade-offs that future studies with larger cohorts and functional endpoints could address. Nevertheless, these results enhance our understanding of OA spatial progression and highlight the limited structural efficacy of ASU under these conditions, guiding future preclinical research. Importantly, ongoing refinement and ethical optimization of animal experimentation, following the 3R principles (replacement, reduction, and refinement), continue to be essential for responsibly advancing translational osteoarthritis research.

## Data Availability

The original contributions presented in the study are included in the article/Supplementary material, further inquiries can be directed to the corresponding author.

## Footnotes

1
https://eda.nc3rs.org.uk/
