# Supplementary material for: 27-Week avocado-soybean unsaponifiables treatment in a knee osteoarthritis rabbit model: histomorphometric assessment of potential disease-modifying effects
Source: Front Vet Sci. 2026 May 29;13:1821515. doi: 10.3389/fvets.2026.1821515 (PMC13261705; doi:10.3389/fvets.2026.1821515)
Supplement: Supplementary file 2 [file Table_1.docx]

| **Femoral condyles** | | **Zones** | **nCg.Th (µm)** | | **cCg.Th (µm)** | | **Cg.Th (µm)** | | **SB.Th (µm)** | |
| --- | --- | --- | --- | --- | --- | --- | --- | --- | --- | --- |
|  |  |  | **Lateral** | **Medial** | **Lateral** | **Medial** | **Lateral** | **Medial** | **Lateral** | **Medial** |
| Osteoarthritis joints (OA) | SHAM | Total | **350.05±95.88º*** | | 144.53±45.05 | | **487.02±110.76º*** | | **310.10±109.44*** | |
|  |  | 1 | 308.90±94.56 | 330.86±98.77 | 155.99±64.04 | 165.52±41.11 | 462.96±116.13 | 495.34±125.70 | 301.97±98.78 | 248.61±81.90 |
|  |  | 2 | 324.88±74.29 | 365.82±80.27 | 153.15±28.41 | 193.63±47.69 | 474.91±90.49 | 557.12±91.03 | 369.57±104.41 | 301.15±68.37 |
|  |  | 3 | **329.37±94.67º*** | 415.71±99.98 | 120.18±22.59 | 137.74±28.03 | **446.10±103.23º*** | 506.50±132.71 | 413,79±118,01 | 304.62±79.20 |
|  |  | 4 | **318.09±99.83º** | 406.77±103.82 | 105.39±27.51 | 124.60±33.33 | **423.14±106.67º** | 530.56±105.02 | 283.75±144.56 | 257.29±106.32 |
|  | CONT | Total | **478.13±164.10ª^** | | 154.86±61.95 | | **625.09±201.56ª^** | | 288.28±111.57 | |
|  |  | 1 | 519.96±206.74 | 481.86±98.77 | 156.13±57.80 | 178.30±55.83 | **679.75±240.84^** | 658.65±125.07 | 217.47±83.42 | 258.79±83.72 |
|  |  | 2 | 500.51±221.56 | 453.46±128.61 | 182.98±69.47 | 172.88±47.62 | 685.02±278.19 | 620.49±185.88 | 272.01±102.62 | 273.80±30.31 |
|  |  | 3 | **483.63±119.97ª^** | 421.07±164.20 | 159.25±73.16 | 159.25±73.16 | **638.71±179.83ª^** | 530.08±174.27 | 363,46±161,16 | 351.25±143.71 |
|  |  | 4 | **576.74±147.42ª*^** | 390.96±176.58 | 139.42±39.04 | **94.20±24.14*** | **714.92±153.58ª^** | 480.58±191.04 | 243.24±76.41 | 324.19±108.92 |
|  | ASU | Total | **436.63±125.47ª^** | | 159.80±61,02 | | **598.84±158.21ª^** | | **226.39±89.193ª^** | |
|  |  | 1 | 297.28±74.19 | 481.48±126.99 | 130.97±52.62 | 128.45±32.01 | 437.49±136.72 | 622.26±212.93 | **165.85±30.99ª** | 205.52±94.94 |
|  |  | 2 | 494.43±118.32 | 461.00±173.42 | 192.35±88.88 | 127.85±36.94 | 683.51±204.57 | 592.24±190.07 | 232.45±64.44 | 220.57±48.21 |
|  |  | 3 | **499.72±95.76ª^** | 480.73±55.65 | 206.80±63.60 | 181.15±46.74 | **707.56±82.42ª^** | 663.63±79.94 | 273,70±125.45 | 265.13±152.66 |
|  |  | 4 | **364.15±78.13º** | 431.46±24.80 | 153.09±77.64 | **169.24±67.55º** | 524.41±138.24 | 604.06±71.90 | 230.04±45.79 | 223.91±115.55 |
| Healthy joints (HT) | SHAM | Total | **323.70±103.53º*** | | 128.40±41.08 | | **449.05±127.66º*** | | **319.92±100.14*** | |
|  |  | 1 | **187.96±51.80º** | 345.11±92.90 | 93.50±33.56 | 157.47±47.32 | **279.79±81.84º** | 499.57±107.12 | **362.45±52.19*** | 274.93±90.31 |
|  |  | 2 | 298.93±42.93 | 375.19±101.50 | 139.71±35.01 | 179.88±38.21 | 436.23±60.13 | 551.64±132.76 | 409.66±78.87 | 342.94±131.51 |
|  |  | 3 | **275.33±67.19º*** | 415.01±59.71 | **114.84±20.94º*** | 136.18±27.35 | **385.43±77.95º*** | 547.17±83.06 | 296.79±86.45 | 335.86±76.63 |
|  |  | 4 | **273.85±104.86º** | 395.31±94.28 | 91.46±13.61 | 110.76±14.67 | **363.94±107.23º** | 502.66±99.82 | 265.05±101.99 | 290.56±123.87 |
|  | CONT | Total | **333.75±104.67º*** | | 123.81±36.84 | | **452.96±121.28º*** | | **331.51±104.12*** | |
|  |  | 1 | 270.71±112.06 | 366.89±147.44 | 127.27±31.66 | 141.63±36.09 | **392.74±118.67º** | 490.38±150.54 | 332.85±50.50 | 390.12±139.56 |
|  |  | 2 | 337.71±105.37 | 307.72±105.11 | 154.01±51.17 | 145.78±40.26 | 488.72±151.53 | 449.43±135.68 | 363.46±66.21 | 292.63±112.62 |
|  |  | 3 | **311.21±81.48º*** | 389.43±71.77 | 119.68±35.47 | 105.64±20.50 | **427.00±115.35º*** | 489.29±75.63 | 319.07±79.17 | 360.62±129.94 |
|  |  | 4 | **301.61±119.09º** | 378.59±76.43 | 104.97±22.71 | **97.02±20.21*** | **403.24±138.19º** | 468.20±95.37 | 268.44±88.61 | 310.87±108.88 |
|  | ASU | Total | **305.42±95.20º*** | | 131.49±38.64 | | **437.46±121.75º*** | | 294.85±98.42 | |
|  |  | 1 | **176.22±43.97º** | 340.04±18.45 | 104.45±33.30 | 128.98±17.45 | **314.51±120.45º** | 455.49±40.57 | 245.12±141.16 | 332.80±66.65 |
|  |  | 2 | 315.34±100.95 | 389.14±104.93 | 154.77±63.41 | 135.87±26.93 | 468.78±155.82 | 524.39±104.65 | 324.75±89.23 | 267.86±51.85 |
|  |  | 3 | **297.13±76.98º*** | 374.66±88.06 | 147.93±37.41 | 149.59±5.84 | **443.76±106.24*** | 521.79±92.32 | 333.17±153.51 | 319.56±37.90 |
|  |  | 4 | **228.22±88.09º** | 324.43±47.78 | 107.08±37.07 | 119.38±41.23 | **340.45±126.27º** | 445.40±77.96 | 223.91±71.23 | 312.34±112.86 |

Supplementary Material

# Supplementary Tables

**Supplementary Table 1. Histomosphometric data from femoral condyles**. ª vs SHAM OA, º vs CONT OA, * vs ASU OA, ^ vs contralateral HT joint.

| **Tibial plateaus** | | **Zones** | **nCg.Th (µm)** | | **cCg.Th (µm)** | | **Cg.Th (µm)** | | **SB.Th (µm)** | |
| --- | --- | --- | --- | --- | --- | --- | --- | --- | --- | --- |
|  |  |  | **Lateral** | **Medial** | **Lateral** | **Medial** | **Lateral** | **Medial** | **Lateral** | **Medial** |
| **Osteoarthritis joints (OA)** | **SHAM** | **Total** | **489.85±257.26º** | | **89.76±27.08*** | | **576.61±252.33º** | | 383.83±113.98 | |
|  |  | **1** | 477.85±87.59 | 651.52±239.86 | 132.71±27.66 | 91.97±24.13 | 610.60±82.71 | 739.17±236.18 | 291.82±186.55 | 414.62±51.35 |
|  |  | **2** | 516.90±88.22 | 816.82±284.96 | **89.06±20.43*** | 71.08±7.05 | 600.96±75.35 | 878.18±275.86 | 330.44±122.69 | 444.21±84.94 |
|  |  | **3** | **273.57±42.13º** | 658.05±203.22 | 79.90±10.86 | 72.52±19.39 | **350.29±43.56º*** | 731.08±196.87 | 397.87±65.10 | 452.03±82.05 |
|  |  | **4** | 154.02±09.43 | 370.04±139.92 | 80.32±20.40 | 100.53±27.12 | 232.41±32.61 | 470.20±139.74 | 359.35±116.05 | 380.29±100.13 |
|  | **CONT** | **Total** | **644.06±260.53ª^** | | **99.44±52.85*** | | **741.20±250.92ª^** | | 322.87±130.06 | |
|  |  | **1** | 593.95±310.96 | 782.83±279.39 | 137.28±60.05 | 82.45±29.86 | 735.34±329.55 | 866.94±253.20 | 264.83±147.09 | 448.53±163.53 |
|  |  | **2** | 656.44±186.22 | 974.09±97.24 | **120.49±49.42*** | 57.11±10.70 | **772.27±173.35^** | 1024.82±97.94 | 304.52±144.84 | 326.20±131.42 |
|  |  | **3** | **529.05±152.64ª^** | 740.43±188.85 | 133.91±85.47 | 61.51±21.26 | **668.31±97.68ª^** | 796.81±126.00 | 280.90±91.56 | 325.00±93.13 |
|  |  | **4** | **362.98±222.87^** | 553.92±125.38 | 90.03±34.99 | 107.46±35.75 | 450.37±246.42 | 650.23±131.01 | 274.99±85.93 | 358.37±113.41 |
|  | **ASU** | **Total** | **565.04±315.46^** | | **145.08±70.94ªº** | | **718.94±324.21^** | | **323.47±138.24^** | |
|  |  | **1** | 361.20±175.9 | 659.63±336.09 | 147.20±67.69 | 155.55±120.92 | 505.08±180.57 | 851.79±295.82 | 195.40±144.71 | 404.39±141.70 |
|  |  | **2** | 519.76±145.19 | 791.23±147.26 | **179.12±46.56ªº^** | 113.63±81.29 | **700.34±123.74^** | 886.57±102.04 | 246.26±86.29 | 424.84±105.86 |
|  |  | **3** | 431.34±151.16 | 647.39±27.17 | 141.10±31.00 | 131.13±101.07 | **598.78±175.46ª^** | 796.81±209.01 | 282.86±43.09 | 409.38±148.36 |
|  |  | **4** | 632.96±699.67 | 579.84±120.45 | 120.19±50.76 | 174.87±109.94 | 775.05±744.27 | 748.19±219.52 | 312.90±174.92 | 421.99±71.67 |
| **Healthy joints (HT)** | **SHAM** | **Total** | **497.22±283.46º** | | **85.23±29.14*** | | **579.12±283.03º** | | **400.88±138.69º** | |
|  |  | **1** | 421.83±95.72 | 718.42±165.38 | 133.76±32.92 | 87.53±14.26 | 553.27±88.79 | 808.22±166.26 | 314.06±201.40 | 493.08±86.47 |
|  |  | **2** | 436.50±128.13 | 1034.55±113.86 | **93.74±19.75*** | 69.89±13.39 | **525.42±130.22º** | 1104.40±112.50 | 451.93±182.85 | 465.00±69.68 |
|  |  | **3** | **238.12±76.55º** | 682.82±54.87 | **76.53±23.42*** | 63.23±19.50 | **308.54±89.78º*** | 737.33±50.50 | 354.31±100.12 | 379.29±58.79 |
|  |  | **4** | 145.04±20.02 | 425.05±67.06 | 67.50±10.37 | 84.55±16.23 | 208.44±29.61 | 507.48±59.71 | 375.74±170.45 | 391.58±103.88 |
|  | **CONT** | **Total** | **462.02±254.79º** | | **89.62±30.24*** | | **548.15±244.70º** | | 369.73±126.33 | |
|  |  | **1** | 419.67±151.53 | 767.16±107.28 | 127.94±31.99 | 71.07±9.27 | 545.38±136.55 | 840.47±96.31 | 282.98±153.45 | 388.80±119.23 |
|  |  | **2** | 440.49±182.26 | 845.86±108.91 | **94.44±19.75*** | 66.39±23.98 | **527.80±167.95º** | 904.53±103.87 | 333.04±96.62 | 436.11±70.94 |
|  |  | **3** | **249.11±138.75º** | 594.94±107.93 | 79.75±10.10 | 80.84±31.63 | **322.68±135.03º*** | 672.21±104.05 | 387.29±121.79 | 396.04±111.32 |
|  |  | **4** | **141.05±44.82º** | 366.88±84.21 | 83.28±15.92 | 106.79±39.36 | 223.34±41.38 | 472.43±65.06 | 312.56±135.44 | 447.05±134.39 |
|  | **ASU**  **Supplementary Table 2. Histomosphometric data from tibial plateaus.** ª vs SHAM OA, º vs CONT OA, * vs ASU OA, ^ vs contralateral HT joint. | **Total** | **378.66±281.90º*** | | **124.99±56.77ªº** | | **509.38±291.40º*** | | **440.30±130.84º*** | |
|  |  | **1** | 220.83±161.32 | 664.76±594.10 | 119.78±55.59 | 106.25±46.15 | 340.44±212.91 | 764.13±553.01 | 316.78±122.71 | 474.79±138.06 |
|  |  | **2** | **234.38±142.** **44º** | **472.86±156.49º** | **107.34±30.10*** | **144.41±90.99º** | **336.48±179.10º*** | 676.60±162.15 | 407.44±159.53 | 489.15±94.22 |
|  |  | **3** | **236.37±93.11º** | **432.85±114.85º** | 101.50±48.46 | **147.88±50.19º** | **338.22±137.00º*** | **576.68±83.65º** | 404.66±120.23 | 506.25±81.69 |
|  |  | **4** | 342.84±296.72 | 364.02±151.61 | 108.09±42.49 | 160.07±72.44 | 450.21±314.90 | 523.91±213.54 | 398.03±190.15 | 494.05±95.25 |

| **FI** | | **Osteoarthritis joints (OA)** | | | **Healthy joints (HT)** | | |
| --- | --- | --- | --- | --- | --- | --- | --- |
|  |  | **SHAM** | **CONT** | **ASU** | **SHAM** | **CONT** | **ASU** |
| **FEMUR** | **Lateral** | **1.05±0.04*** | **1.06±0.05*** | **1.21±0.21ªº** | **1.03±0.02*** | **1.03±0.02*** | **1.09±0.04ª** |
|  | **Medial** | **1.07±0.07*** | **1.19±0.25^** | **1.13±0.05ª** | **1.06±0.04*** | **1.04±0.04º*** | 1.11±0.07 |
| **TIBIA** | **Lateral** | **1.02±0.05º*** | **1.11±0.15ª** | **1.31±0.46ª** | **1.02±0.03º*** | **1.02±0.02*** | **1.16±0.37ª** |
|  | **Medial** | 1.20±0.25 | 1.35±0.30 | 1.30±0.58 | 1.23±0.13 | 1.22±0.33 | **1.13±0.18º** |

**Supplementary Table 3. Fibrillation index data**. ª vs SHAM OA, º vs CONT OA, * vs ASU OA, ^ vs contralateral HT joint.

| **TB** | | **Osteoarthritis joints (OA)** | | | **Healthy joints (HT)** | | |
| --- | --- | --- | --- | --- | --- | --- | --- |
|  |  | **SHAM** | **CONT** | **ASU** | **SHAM** | **CONT** | **ASU** |
| **Tb.A (%)** | **LF** | 39.91±8.46 | 50.49±11.84 | 42.59±5.29 | 46.76±6.90 | 45.62±5.77 | 45.20±4.27 |
|  | **MF** | 45.32±3.77 | 43.53±5.91 | 39.78±8.24 | 50.95±9.80 | 48.70±8.79 | 46.50±5.92 |
|  | **LT** | **63.83±5.80*** | 51.91±10.04 | **42.46±3.21ª** | **62.69±5.36*** | **61.62±2.46*** | 44.22±13.88 |
|  | **MT** | 48.51±10.10 | 46.53±16.25 | 42.51±10.05 | 51.40±10.22 | 56.42±7.63 | 43.58±8.60 |
| **Tb.Th (mm)** | **LF** | 0.43±0.13 | 0.49±0.31 | 0.44±0.08 | 0.66±0.96 | 0.34±0.14 | 0.33±0.08 |
|  | **MF** | 0.43±0.18 | 0.45±0.21 | 0.30±0.08 | 0.48±0.26 | 0.47±0.22 | 0.47±0.31 |
|  | **LT** | 0.30±0.08 | 0.26±0.09 | 0.32±0.08 | 0.37±0.15 | 0.33±0.06 | 0.33±0.04 |
|  | **MT** | 0.34±0.06 | 0.48±0.25 | 0.40±0.20 | 0.33±0.07 | 0.34±0.11 | 0.43±0.12 |
| **Tb.Sp (mm)** | **LF** | 0.51±0.23 | 0.48±0.19 | 0.48±0.12 | 0.43±0.20 | 0.36±0.13 | 0.45±0.20 |
|  | **MF** | 0.52±0.29 | 0.44±0.20 | 0.44±0.13 | 0.50±0.18 | 0.39±0.22 | 0.34±0.14 |
|  | **LT** | 0.22±0.12 | 0.30±0.09 | 0.32±0.03 | 0.18±0.08 | 0.22±0.07 | 0.31±0.09 |
|  | **MT** | 0.43±0.26 | 0.45±0.22 | 0.55±0.52 | 0.44±0.16 | 0.24±0.08 | 0.24±0.06 |
| **Tb.N (1/mm)** | **LF** | 1.17±0.44 | 1.07±0.22 | 1.10±0.15 | 1.17±0.44 | 1.52±0.39 | 1.37±0.42 |
|  | **MF** | 1.13±0.33 | 1.28±0.44 | 1.44±0.38 | 1.13±0.33 | 1.24±0.31 | 1.46±0.51 |
|  | **LT** | 1.95±0.33 | 1.83±0.30 | 1.60±0.26 | 1.95±0.57 | 1.83±0.30 | 1.60±0.26 |
|  | **MT** | 1.42±0.42 | **1.12±0.24^** | 1.32±0.52 | 1.34±0.19 | **1.92±0.42º** | 1.51±0.21 |

**Supplementary Table 4. Trabecular subchondral bone measurements**. ª vs SHAM OA, º vs CONT OA, * vs ASU OA, ^ vs contralateral HT joint.
